# Disrupted Renal Mitochondrial Homeostasis after Liver Transplantation in Rats

**DOI:** 10.1371/journal.pone.0140906

**Published:** 2015-10-19

**Authors:** Qinlong Liu, Yasodha Krishnasamy, Hasibur Rehman, John J. Lemasters, Rick G. Schnellmann, Zhi Zhong

**Affiliations:** 1 Department of Drug Discovery & Biomedical Sciences, Medical University of South Carolina, Charleston, South Carolina, United States of America; 2 The Second Affiliated Hospital of Dalian Medical University, Dalian, Liaoning Province, China; 3 Department of Biochemistry & Molecular Biology, Medical University of South Carolina, Charleston, United States of America; 4 Ralph H. Johnson Veterans Affairs Medical Center, Charleston, South Carolina, United States of America; The University of Hong Kong, HONG KONG

## Abstract

**Background:**

Suppressed mitochondrial biogenesis (MB) contributes to acute kidney injury (AKI) after many insults. AKI occurs frequently after liver transplantation (LT) and increases mortality. This study investigated whether disrupted mitochondrial homeostasis plays a role in AKI after LT.

**Methods:**

Livers were explanted from Lewis rats and implanted after 18 h cold storage. Kidney and blood were collected 18 h after LT.

**Results:**

In the kidney, oxidative phosphorylation (OXPHOS) proteins ATP synthase-β and NADH dehydrogenase-3 decreased 44% and 81%, respectively, with marked reduction in associated mRNAs. Renal PGC-1α, the major regulator of MB, decreased 57% with lower mRNA and increased acetylation, indicating inhibited synthesis and suppressed activation. Mitochondrial transcription factor-A, which controls mtDNA replication and transcription, protein and mRNA decreased 66% and 68%, respectively, which was associated with 64% decreases in mtDNA. Mitochondrial fission proteins Drp-1 and Fis-1 and mitochondrial fusion protein mitofusin-1 all decreased markedly. In contrast, PTEN-induced putative kinase 1 and microtubule-associated protein 1A/1B-light chain 3 increased markedly after LT, indicating enhanced mitophagy. Concurrently, 18- and 13-fold increases in neutrophil gelatinase-associated lipocalin and cleaved caspase-3 occurred in renal tissue. Both serum creatinine and blood urea nitrogen increased >2 fold. Mild to moderate histological changes were observed in the kidney, including loss of brush border, vacuolization of tubular cells in the cortex, cast formation and necrosis in some proximal tubular cells. Finally, myeloperoxidase and ED-1 also increased, indicating inflammation.

**Conclusion:**

Suppression of MB, inhibition of mitochondrial fission/fusion and enhancement of mitophagy occur in the kidneys of recipients of liver grafts after long cold storage, which may contribute to the occurrence of AKI and increased mortality after LT.

## Introduction

Orthotopic liver transplantation (LT) is the only proven therapy for end-stage liver diseases [1–5]. However, acute renal dysfunction and chronic renal diseases often occur after LT [6–9]. The incidence of perioperative acute kidney injury (AKI) in liver transplant recipients varies significantly, ranging from 17% to 95% [7,9–13]. After LT, 5–30% of recipients have to receive renal replacement therapy due to severe AKI [7,11]. AKI also increases infection, sepsis, and acute rejection and substantially decreases patient survival after LT [11,14–16]. Increasing evidence indicates that AKI also adversely affects long-term patient outcomes [17,18]. Ultimately, acute renal dysfunction in LT recipients prolongs stays in intensive care units and the hospital, and increases re-hospitalization, the need for postoperative dialysis, and the cost of care.

While AKI after LT frequently presents as acute tubular necrosis (ATN, ~70% of AKI) [11,12,14], the mechanisms underlying AKI after LT remain unclear. More severe liver dysfunction and higher MELD scores before transplantation, severe hypotension/hypoperfusion, anesthesia, transfusion of highly packed red blood cells during surgery, and use of calcinurin inhibitors after transplantation may increase the risk of post-transplantation acute renal dysfunction [11,19,20]. Whether the presence of pre-transplantation AKI increases post-transplantation AKI remains controversial [11]. The degree of liver graft dysfunction is a strong and consistent predictor of AKI after LT [11,19,20].

The renal tubular cells have high energy consumption due to active energy-dependent processes such as reabsorption of filtered blood components and secretion of many substances in these cells. Therefore, mitochondrial homeostasis is crucial for proper renal function. Mitochondrial homeostasis is maintained by mitochondrial biogenesis (MB), mitophagy and mitochondrial dynamics, and disrupted mitochondrial homeostasis frequently leads to organ failure [21]. Persistent disruption of mitochondrial homeostasis has been observed in several animal models of AKI [21,22]. MB is a process that generates new mitochondria in response to increased energy demand (e.g. exercise) and mitochondrial stress/damage [23]. Suppression of MB reduces the capability of cells to adapt to stresses and to maintain proper mitochondrial function, increasing injury and/or inhibiting functional recovery and repair processes after injury. In recent years, evidence suggests that inhibited MB and mitochondrial dysfunction play essential roles in AKI caused by many different insults. For example, renal MB suppression occurs after kidney ischemia/reperfusion (I/R), sepsis, folic acid and glycerol treatment, leading to decreased oxidative phosphorylation (OXPHOS) proteins, mitochondrial dysfunction and renal injury [22,24–28]. In contrast, stimulation of MB attenuates AKI [22,24–28]. Mitophagy selectively removes depolarized/damaged mitochondria, thus controlling mitochondrial quality [29,30]. Inhibited mitophagy leads to impairment of mitochondrial function [31]. Mitochondria divide (fission) and fuse (fusion) continuously in healthy cells [32]. Mitochondrial dynamics also play an essential role in mitochondrial quality control, thus affecting cell function and survival [33–35]. Alteration of mitochondrial dynamics occurs in I/R- and glycerol-induced AKI [22]. Whether mitochondrial homeostasis is disrupted in the kidney after LT and its relation to occurrence of post-transplantation AKI remains unknown. Therefore, in the present study we explored renal MB, mitophagy and mitochondrial dynamics after LT.

## Materials and Methods

### Rat liver transplantation

Inbred male Lewis rats (200–250 g) were used as both donors and recipients in LT experiments. LT was performed under isofluorane anesthesia (2–3%) using the two-cuff technique with the hepatic artery and bile duct re-constructed as described previously [36]. Liver grafts were stored in University of Wisconsin storage solution (Bridge to Life, Ltd., Columbia, SC) at 0–1°C for 18 hours. Anhepatic time was ~17 min, and implantation surgery took ~40 min. For sham operation, ligaments around the liver were dissected, and the abdominal wall was closed 40 min later without transplantation.

### Blood creatinine and urea nitrogen

Under pentobarbital anesthesia (50 mg/kg, ip) at 18 h after implantation, blood samples were collected from the inferior vena cava. Serum was obtained by centrifugation. Serum creatinine and blood urea nitrogen (BUN) were determined using analytical kits from Sigma-Aldrich (St. Louis, MO) and Bioassay Systems (Atlanta, GA), respectively, according to the manufacturers’ protocols.

### Histology

Kidneys were collected 18 h after implantation under pentobarbital anesthesia and fixed with 4% paraformaldehyde (VWR Inc., West Chester, PA) in 0.1 mM phosphate buffered-saline (Mediatech Inc., Manassas, VA). Tissue blocks were imbedded in paraffin after 48-hour fixation. After hematoxylin-eosin (H&E) staining, kidney sections were analyzed microscopically for pathology (Zeiss Axiovert 100 microscope, Thornwood, NY) using a 20x objective lens [37].

### Analysis of mitochondrial DNA (mtDNA) content

mtDNA copy number was assessed by quantitative polymerase chain reaction (qPCR) [38,39]. Total DNA was isolated from renal cortex using the DNeasy Blood and Tissue Kit (Qiagen, Valencia, CA) and mtDNA content was determined as mtDNA-encoded NADH dehydrogenase-1 and normalized against the nuclear-encoded POU class 5 homeobox 1 gene as described previously [38,39].

### Detection of the mRNAs of oxidative phosphorylation (OXPHOS) proteins and MB signaling molecules

mRNAs of ATP synthase-β (AS-β), NADH dehydrogenase-3 (ND3), peroxisome proliferator-activated receptor γ co-activator 1α (PGC-1α) and mitochondrial transcription factor A (Tfam) were detected by qPCR as described elsewhere [38,39]. PCR reactions were performed in triplicate with a reaction mixture containing 2x IQ^TM^SYBR Supermix (Bio-Rad), cDNA template, and 0.1 nM of the forward and reverse primers using a CFX96 Real Time-PCR Detection System (Bio-Rad, Hercules, CA). The abundance of mRNA was normalized against hypoxanthine phospho-ribosyl-transferase using the ΔΔ*Ct* method.

### PGC-1α immunoprecipitation (IP)

PGC-1α activity is inhibited by acetylation [40,41]. PGC-1α acetylation status was assessed by immunoblotting of acetylated lysine residues after IP of PGC-1α from kidney cortex homogenates as described previously [38]. Protein content in the immunoprecipitates was measured using the Bio-Rad reagent (Bio-Rad Laboratory, Hercules, CA), an equal amount of PGC-1α (10 μg) was loaded to each lane, and immunoblotting was performed using specific antibody against acetylated lysine residues (Cell Signaling Technology, Danvers, MA) [38].

### Immunoblotting

Proteins of interest in renal cortex homogenates were detected by immunoblotting as described previously [36]. Primary antibodies were against: AS-β, neutrophil gelatinase-associated lipocalin (NGAL), Tfam (GenWay Biotech, San Diego, CA), ED-1 (Serotek, Raleigh, NC), myeloperoxidase (MPO; DAKO, Carpinteria, CA), cleaved caspase-3 (Cell Signaling Technology, Danvers, MA), dynamin-related GTPase protein Drp-1, fissin-1 (Fis-1), mitofusin-1 (Mfn-1), ND3, PGC-1α, PTEN-induced putative kinase 1 (PINK-1) (Santa Cruz Biotech., Santa Cruz, CA) and microtubule-associated protein 1A/1B-light chain 3 (LC3, MBL International, Des Plainers, IL) at concentrations of 1:100 to 1000, and actin (ICN, Costa Mesa, CA) at a concentration of 1:3000. Detection was achieved by chemiluminescence (Pierce Biotechnology, Rockford, IL).

### Statistical analysis

Groups were compared using the Student’s-t test. There were 3–4 rats per group for all parameters. Data shown are means ± S.E.M. Differences were considered significant at p<0.05.

### Ethics statement

All rats were given humane care in compliance with institutional guidelines using protocols approved by the Institutional Animal Care and Use Committee of Medical University of South Carolina. All survival surgeries were performed under isoflurane anesthesia (2–3%) and all non-survival surgeries were performed under sodium pentobarbital anesthesia (50 mg/kg, i.p.).

## Results

### Suppressed renal function after LT

**Fig 1:**
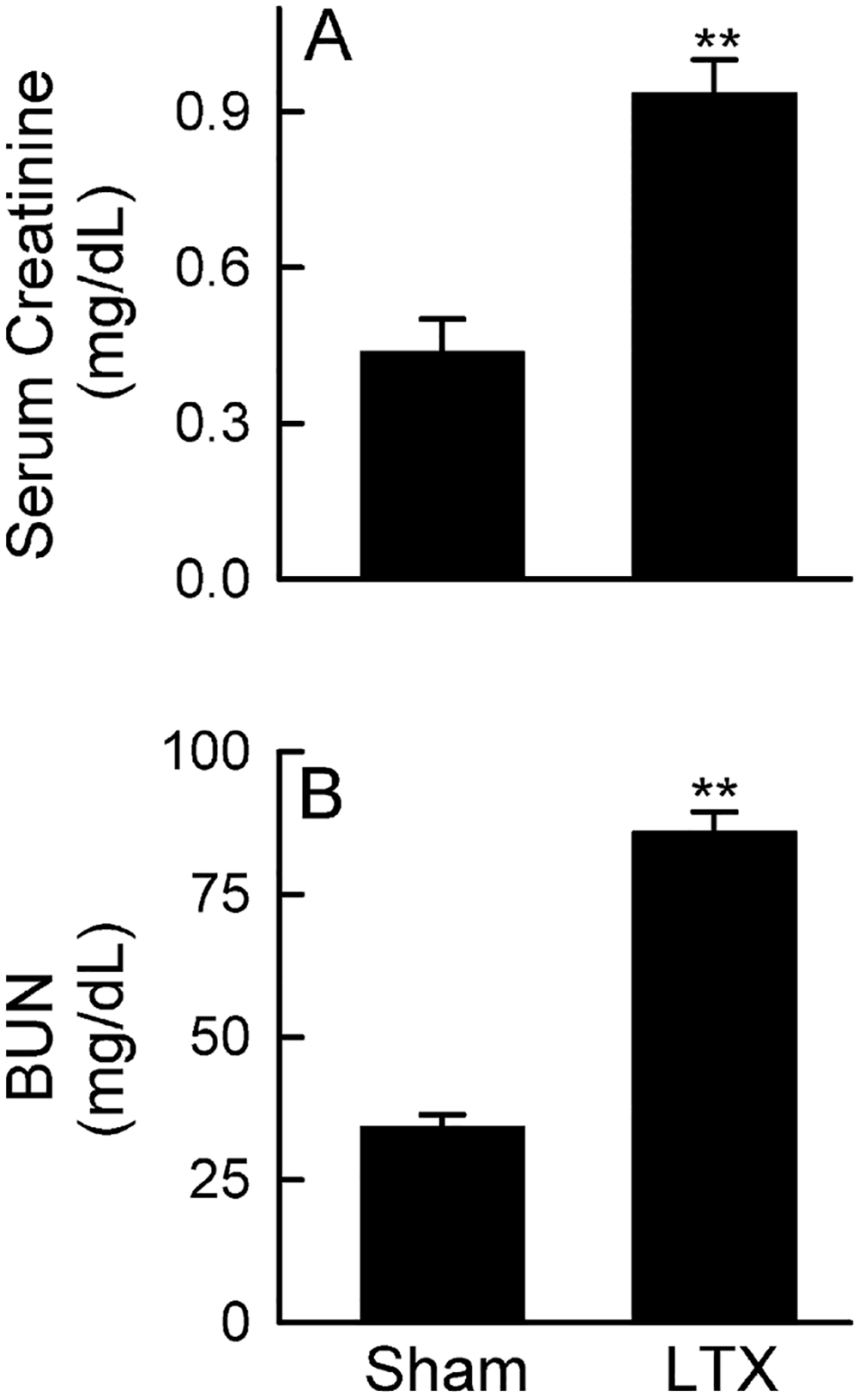
Suppressed renal function after liver transplantation. Transplantation was performed as described in “Methods” and blood was collected 18 h after sham-operation (**Sham**) or liver transplantation (**LTX**). **A**: serum creatinine; **B**: blood urea nitrogen (BUN). Values are mean ± SEM. **p<0.01 vs sham (n = 4/group).

Our previous study showed that transplantation of liver grafts after long preservation caused severe graft injury and decreased survival of recipients to ~25% [36]. Here we investigated whether renal function was altered in these recipients. Serum creatinine increased 2.2 fold and BUN increased 2.5 fold 18 h after LT (Fig 1A and 1B). These results demonstrate that renal dysfunction occurs in the recipients after LT.

### Kidney injury after LT

**Fig 2:**
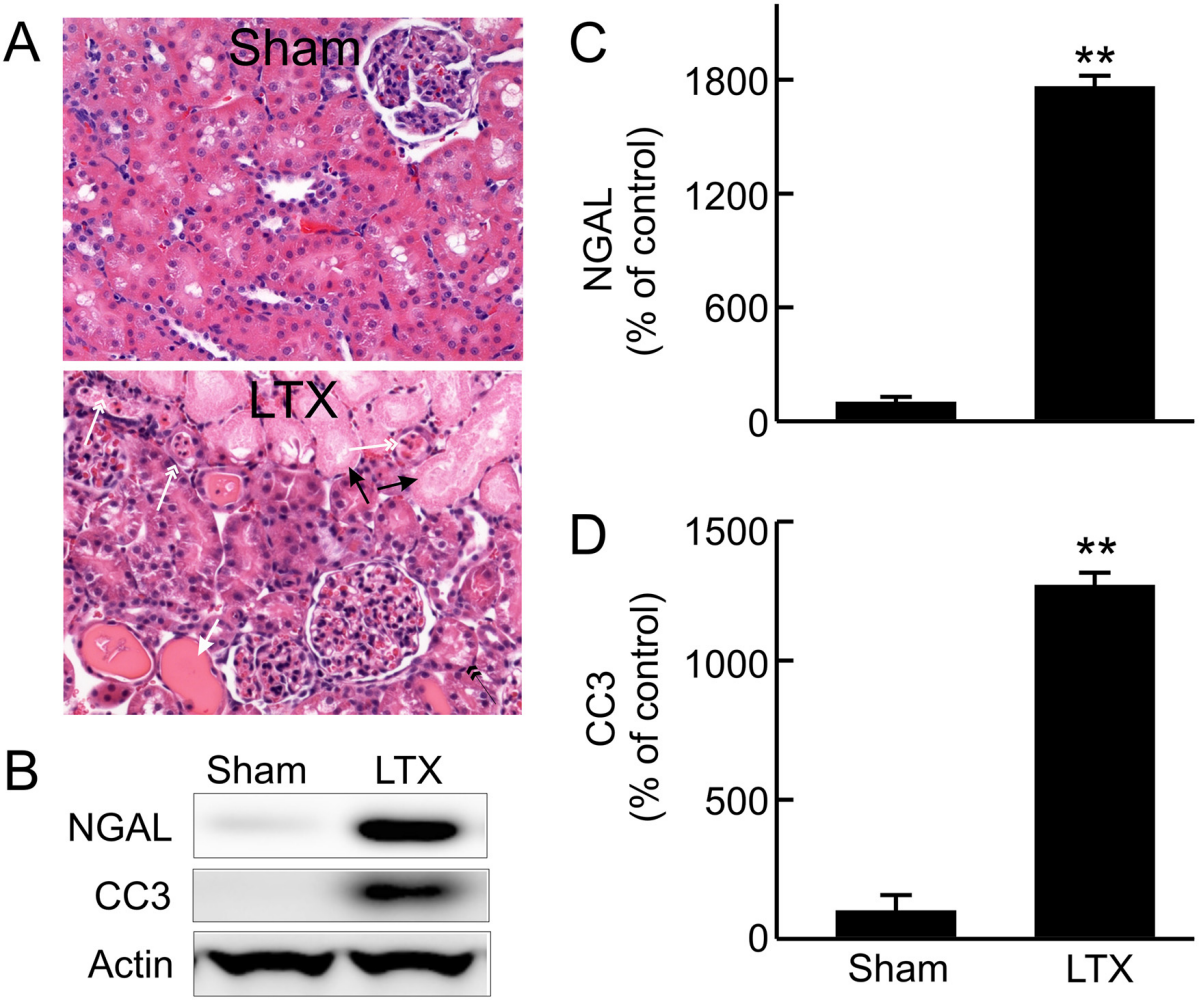
Renal injury after liver transplantation. Transplantation was performed as described in “Methods” and kidneys were collected 18 h after sham-operation (**Sham**) or liver transplantation (**LTX**). **A**, H&E-stained slides; black arrow, necrotic tubules; black double arrow head, loss of brush border; white arrow, hyaline cast formation; white double arrow head, infiltrated leukocytes and other cell debris in tubular lumen. **B**, representative immunoblot images of neutrophil gelatinase-associated lipocalin (NGAL) and cleaved caspase-3 (CC3); **C**, quantification of immunoblot images of NGAL by densitometry; **D**, quantification of immunoblot images of CC3 by densitometry. Values are mean ± SEM. **p<0.01 vs sham (n = 3-4/group).

Renal dysfunction could occur in the presence or absence of overt renal injury. We examined whether pathological changes existed in kidneys of liver recipients (Fig 2A). The kidneys of sham-operated rats showed normal histology. Mild to moderate pathological changes were observed in the kidneys of recipients, mainly loss of brush border and vacuolization of tubular cells in the cortex. Cast formation and necrosis appeared in some proximal tubular cells. Infiltration of leukocytes was also observed in renal tissue and in the casts in tubular lumens. Most of these alterations appeared as patches and located in cortex. No overt pathological changes were observed in glomeruli.

The molecular markers of kidney injury also increased. NGAL, a marker of AKI [42], was barely detectable in the kidneys of sham-operated rats (Fig 2B and 2C). NGAL increased 18 fold after LT. Cleaved caspase-3 was barely detectable in kidneys from sham-operated rats but increased 13-fold after LT (Fig 2B and 2D), indicating apoptosis.

**Fig 3:**
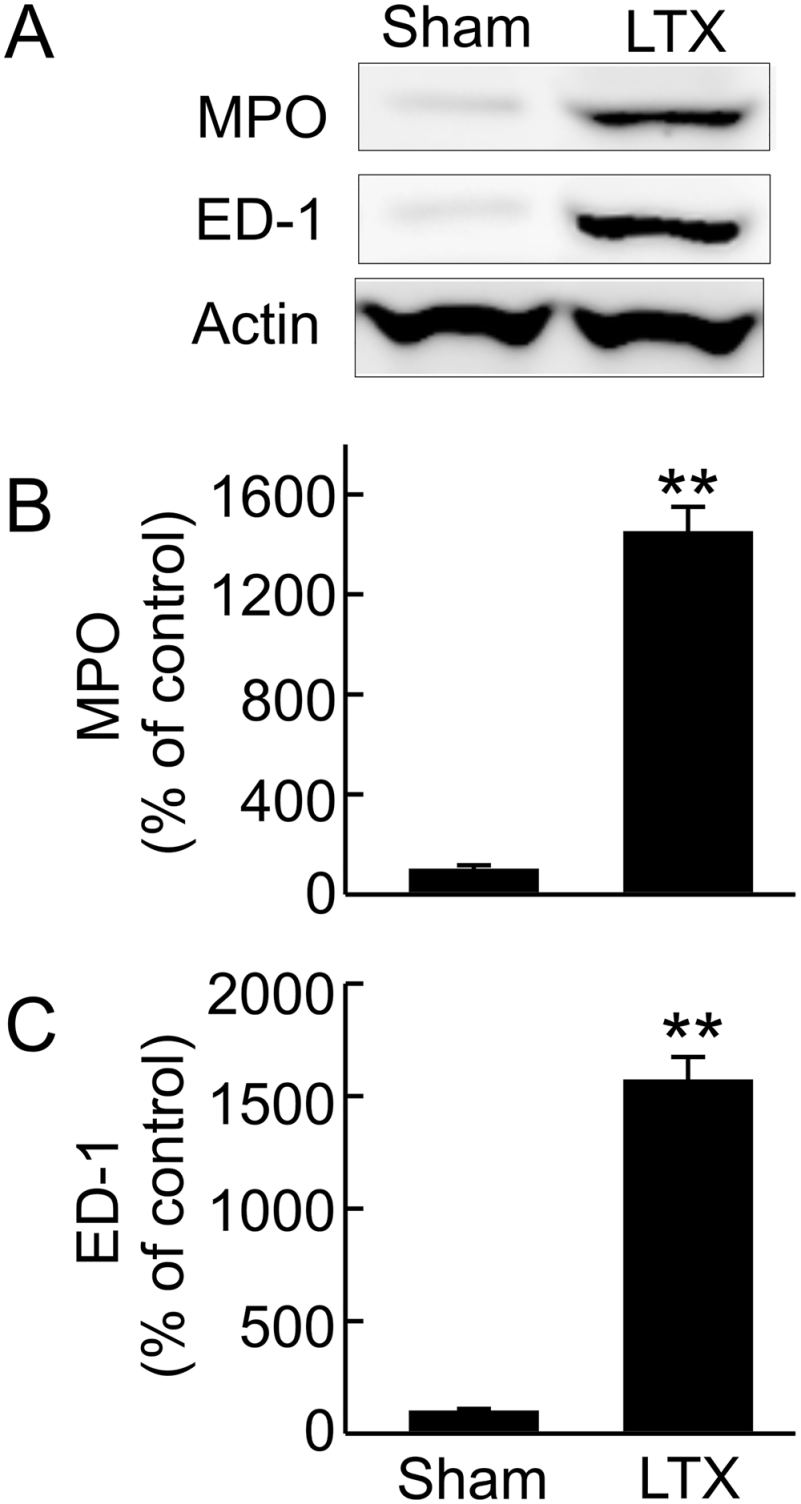
Renal leukocyte infiltration after liver transplantation. Transplantation was performed as described in “Methods” and kidneys were collected 18 h after sham-operation (**Sham**) or liver transplantation (**LTX**). **A**, representative immunoblot images of myeloperoxidase (MPO) and ED-1; **B**, quantification of immunoblot images of MPO by densitometry; **C**, quantification of immunoblot images of ED-1 by densitometry. Values are mean ± SEM. **p<0.01 vs sham (n = 3-4/group).

Consistent with histological leukocyte infiltration, MPO, an indicator of neutrophil infiltration, increased 15-fold after LT (Fig 3A and 3B) and ED-1, a marker of monocyte/macrophage infiltration, increased 16-fold after LT (Fig 3A and 3C).

### Decreases in renal mitochondrial OXPHOS proteins and associated mRNAs after LT

**Fig 4:**
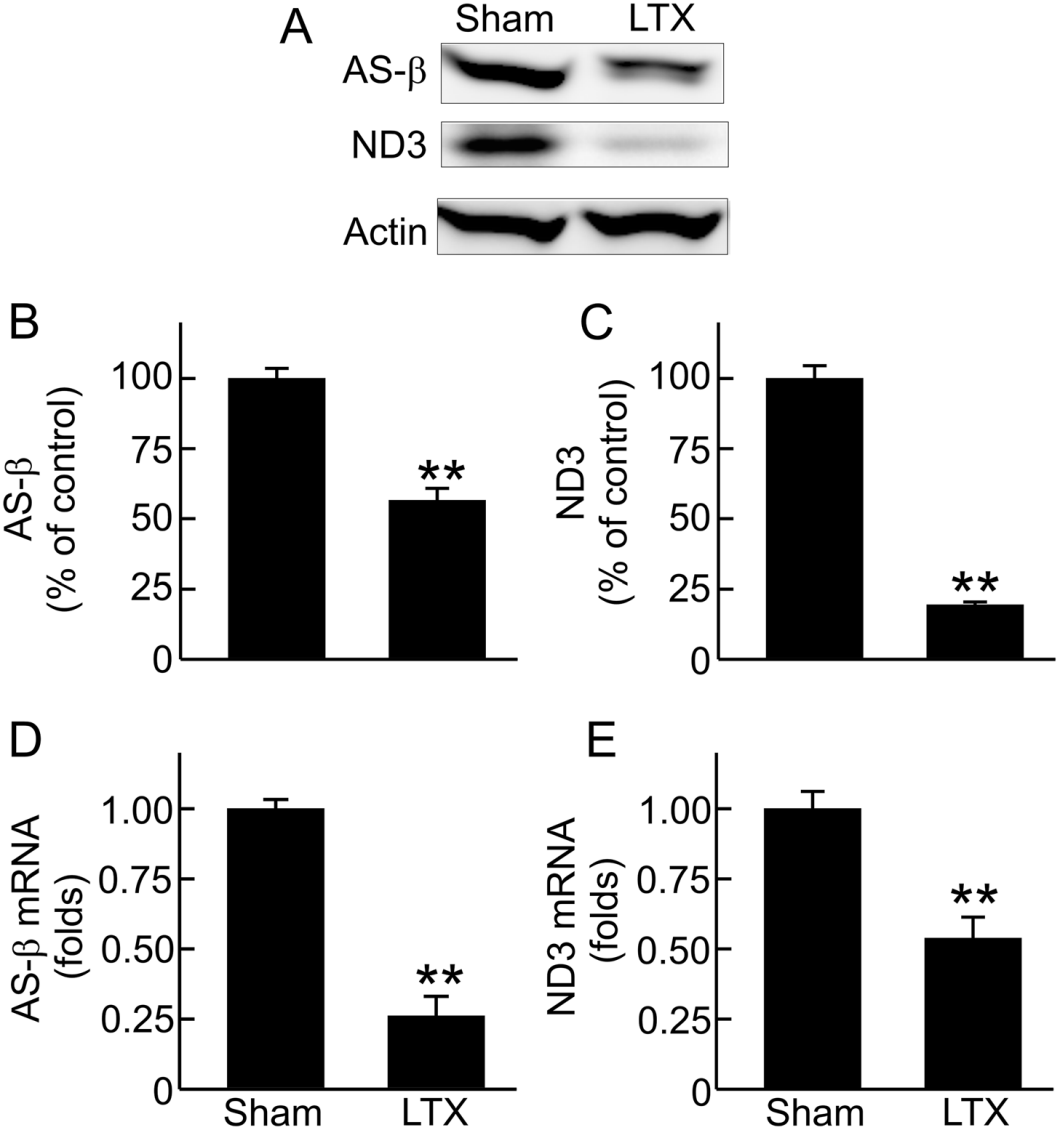
Decreases in renal ATP synthase-β (AS-β) and NADH dehydrogenase-3 (ND3) expression after liver transplantation. Transplantation was performed as described in “Methods” and kidneys were collected 18 h after sham-operation (**Sham**) or liver transplantation (**LTX**). **A**, representative immunoblot images of AS-β and ND3; **B**, quantification of immunoblot images of AS-β by densitometry; **C**, quantification of immunoblot images of ND3; **D**, detection of AS-β mRNA by qPCR; **E**, detection of ND3 mRNA by qPCR. Values are mean ± SEM. **p<0.01 vs sham (n = 3-4/group).

Mitochondrial dysfunction is linked to occurrence of AKI after many acute insults [21,22,24–28]. Decreases in mitochondrial respiratory chain proteins inhibit oxidative phosphorylation and ATP production. To investigate whether mitochondrial homeostasis is disrupted in the kidney after LT, we examined the renal mitochondrial OXPHOS proteins. AS-β, a subunit of mitochondrial respiratory chain Complex V that is encoded by nDNA, decreased 44% compared to sham-operated rats (Fig 4A and 4B). ND3, a mtDNA-encoded mitochondrial OXPHOS protein, decreased 81% (Fig 4A and 4C).

We further measured the associated mRNAs of these proteins to determine whether decreases in these proteins after LT are linked to suppressed formation. AS-β and ND3 mRNAs decreased 74% and 46%, respectively, after LT (Fig 4D and 4E). These data reveal that decreases in AS-β and ND3 proteins are due, at least in part, to decreases in their expression.

### Decreases in renal PGC-1α protein, mRNA and activation after LT

**Fig 5:**
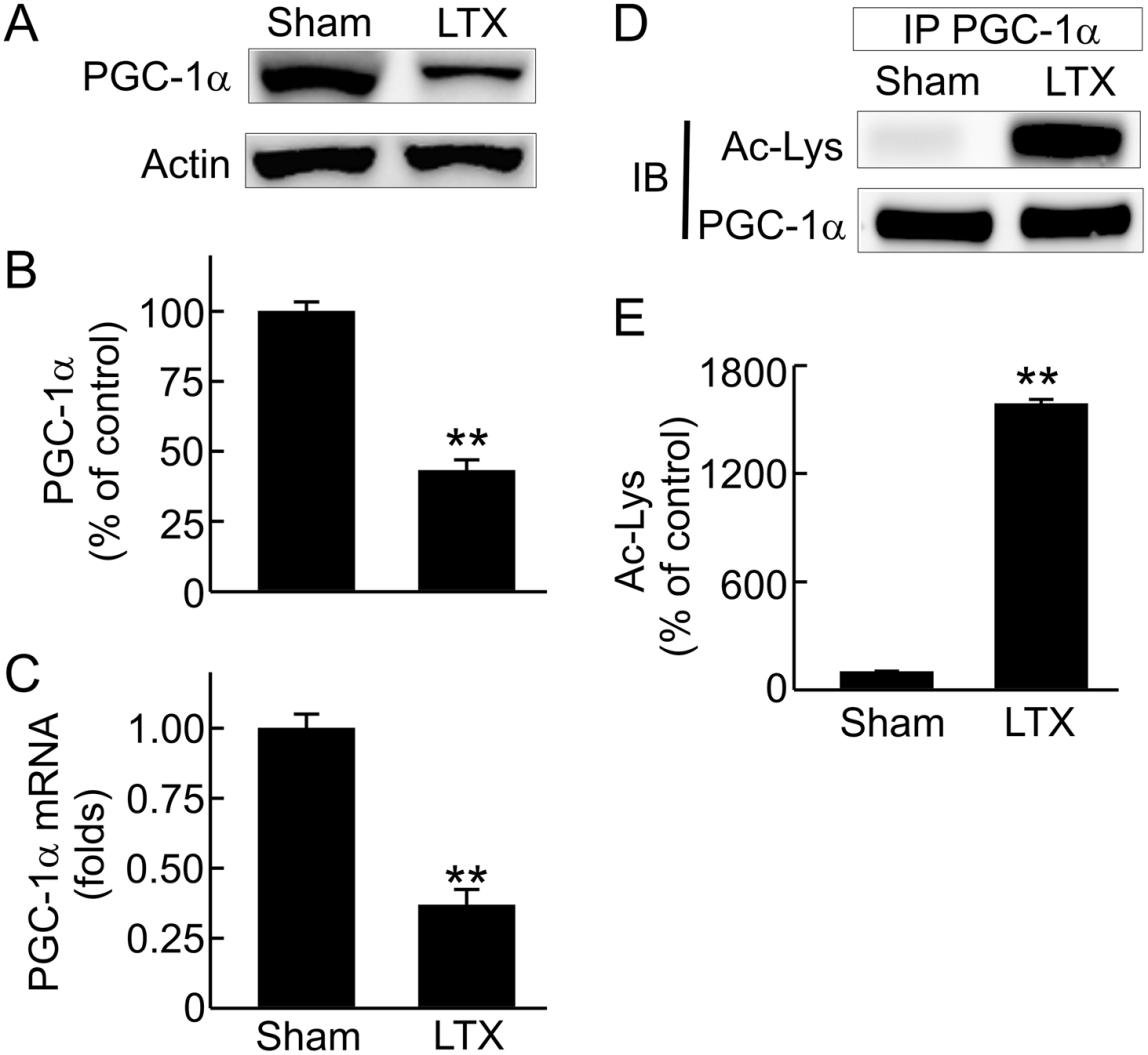
Suppressed renal peroxisome proliferator-activated receptor gamma coactivator 1-alpha (PGC-1α) expression and activation after liver transplantation. Transplantation was performed as described in “Methods” and kidneys were collected 18 h after sham-operation (**Sham**) or liver transplantation (**LTX**). **A**, representative immunoblot images of PGC-1α; **B**, quantification of immunoblot images of PGC-1α by densitometry; **C**, PGC-1α mRNA detected by qPCR; **D**, representative immunoblot images of acetylated lysine residues (Ac-Lys) of immunoprecipitated (IP) PGC-1α; **E**, quantification of immunoblot images of Ac-Lys. Values are mean ± SEM. **p<0.01 vs sham (n = 3-4/group).

MB is primarily regulated by PGC-1α [40]. To explore why MB is altered after LT, we examined PGC-1α in the kidney. PGC-1α decreased 57% in the kidneys of liver recipients compared to sham-operated rats (Fig 5A and 5B). To explore whether decreased PGC-1α is due to suppressed expression, we examined its mRNA. Renal PGC-1α mRNA decreased 63% after LT (Fig 5C). We also investigated whether PGC-1α activation is altered after LT. PGC-1α activity is higher when de-acetylated [39]. PGC-1α acetylation status was detected by immunoblotting of acetylated lysine residuals in immunoprecipitated PGC-1α. After immunoprecipitation, PGC-1α was equally loaded to each lane to avoid the potential influence of decreased PGC-1α protein on detection of acetylation. Acetylated PGC-1α increased markedly after LT (Fig 5D and 5E), indicating suppressed PGC-1α activation.

### Decreased renal Tfam expression and mtDNA copy number after LT

**Fig 6:**
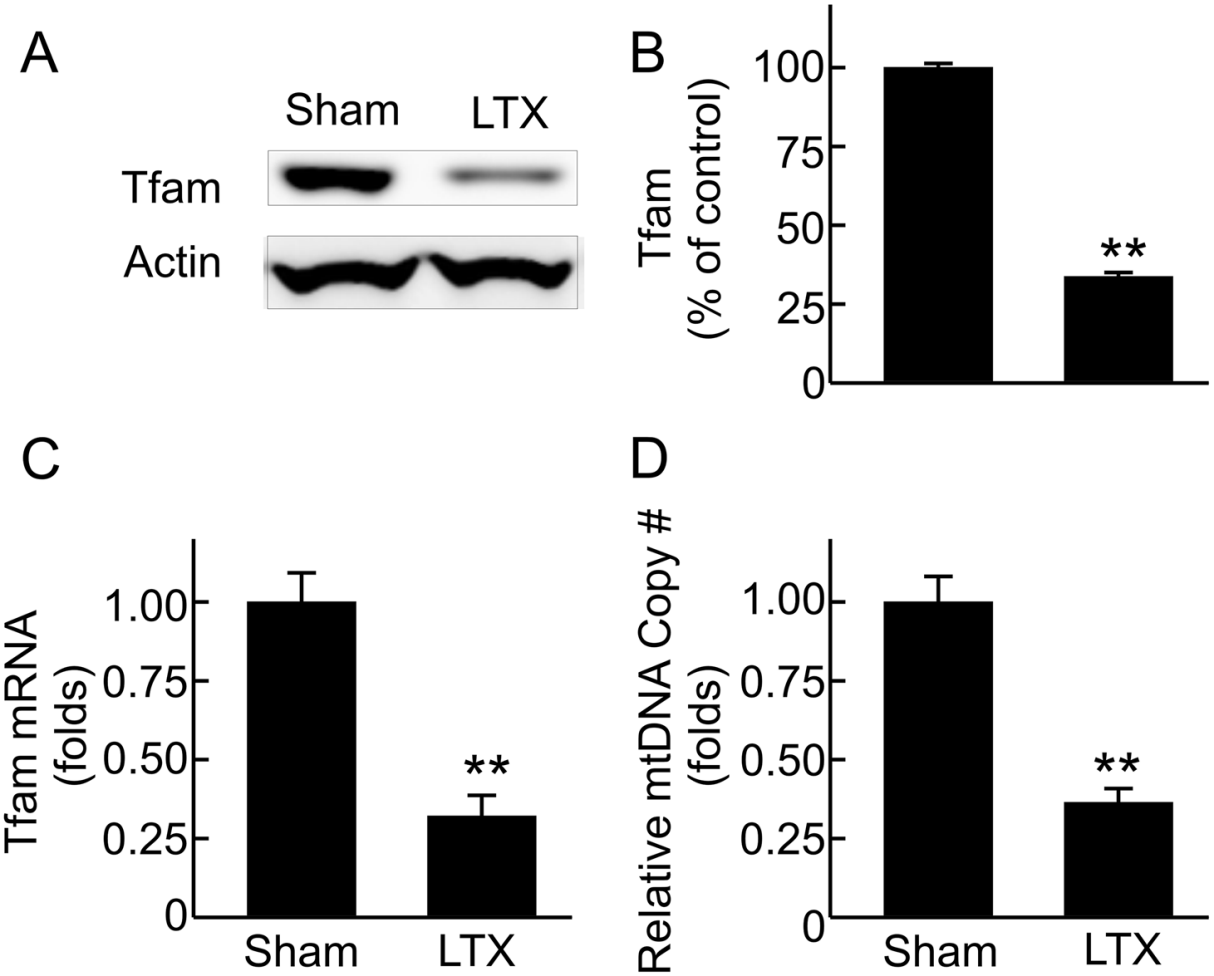
Inhibited renal mitochondrial transcription factor A (Tfam) expression and decreased renal mitochondrial DNA (mtDNA) after liver transplantation. Transplantation was performed as described in “Methods” and kidneys were collected 18 h after sham-operation (**Sham**) or liver transplantation (**LTX**). **A**, representative immunoblot images of Tfam; **B**, quantification of immunoblot images of Tfam by densitometry; **C**, Tfam mRNA detected by qPCR; **D**, relative mitochondrial DNA copy number detected by qPCR. Values are mean ± SEM. **p<0.01 vs sham (n = 3-4/group).

In addition to controlling transcription of nDNA-encoded OXPHOS proteins, PGC-1α modulates expression of Tfam which regulates the replication and transcription of mtDNA [43]. Tfam decreased 66% and Tfam mRNA decreased 68% after LT (Fig 6A, 6B and 6C), consistent with suppressed Tfam expression. Moreover, renal mtDNA copy number decreased 64% after LT (Fig 6D).

### Suppressed renal mitochondrial dynamics after LT

**Fig 7:**
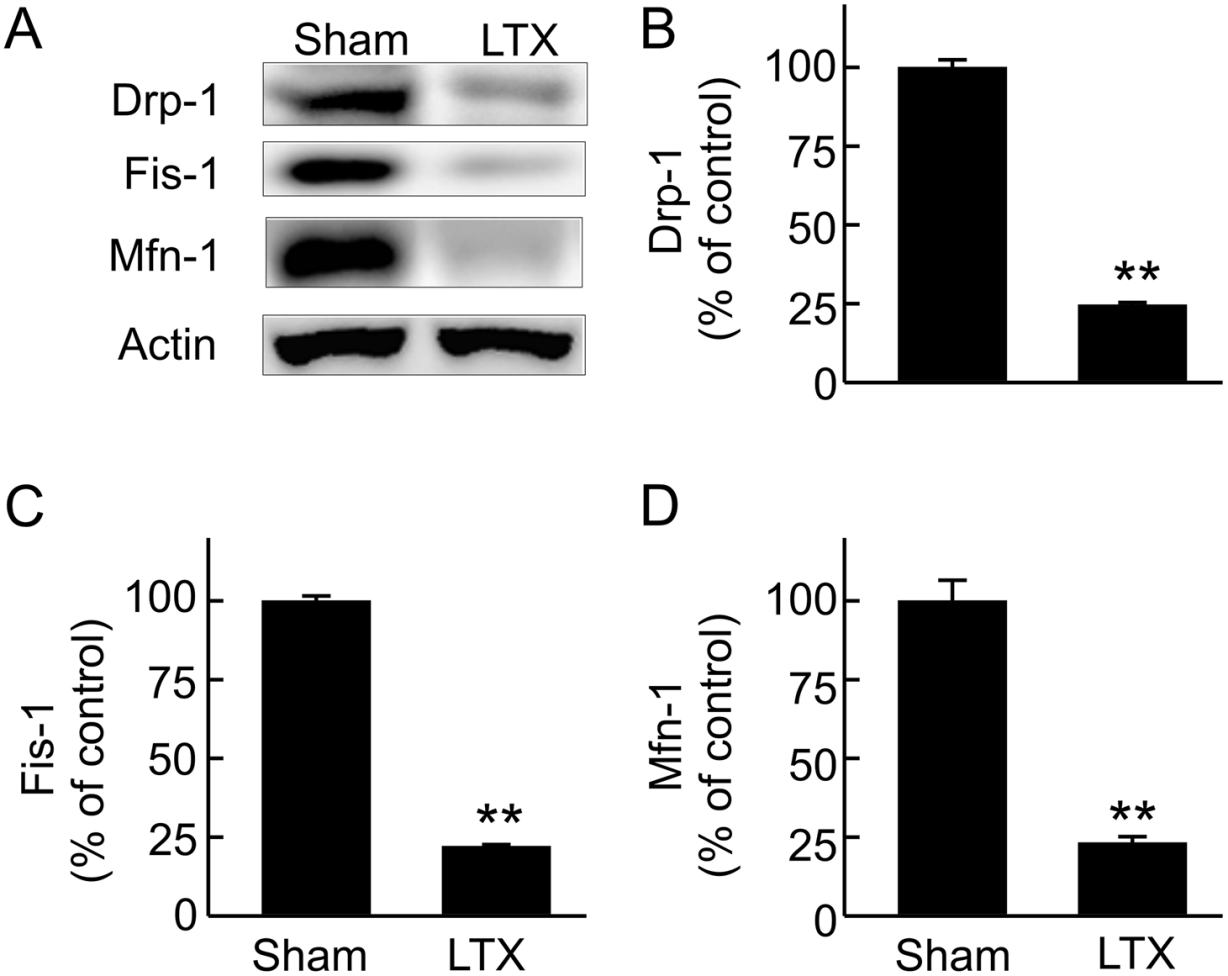
Suppressed renal mitochondrial dynamics after liver transplantation. Transplantation was performed as described in “Methods” and kidneys were collected 18 h after sham-operation (**Sham**) or liver transplantation (**LTX**). **A**, representative immunoblot images of Drp-1, fissin-1 (Fis-1) and mitofusin-1 (Mfn-1); **B**, quantification of immunoblot images of Drp-1 by densitometry; **C**, quantification of immunoblot images of Fis-1 by densitometry; **D**, quantification of immunoblot images of Mfn-1 by densitometry. Values are mean ± SEM. **p<0.01 vs sham (n = 3-4/group).

Mitochondria undergo fission and fusion, and mitochondrial fission is required for MB [44]. We therefore investigated whether mitochondrial dynamic processes are altered after LT. Drp-1, the protein that controls mitochondrial fission, and Fis-1, the protein that recruits Drp-1 to the fission site [45], decreased 75% and 78%, respectively, after LT (Fig 7A, 7B and 7C). Mitofusins (Mfn) mediate mitochondrial fusion [46]. Mfn-1 decreased 77% after LT (Fig 7A and 7D). Together, these data reveal that mitochondrial dynamics are suppressed in the kidney after LT.

### Enhanced renal mitophagy after LT

**Fig 8:**
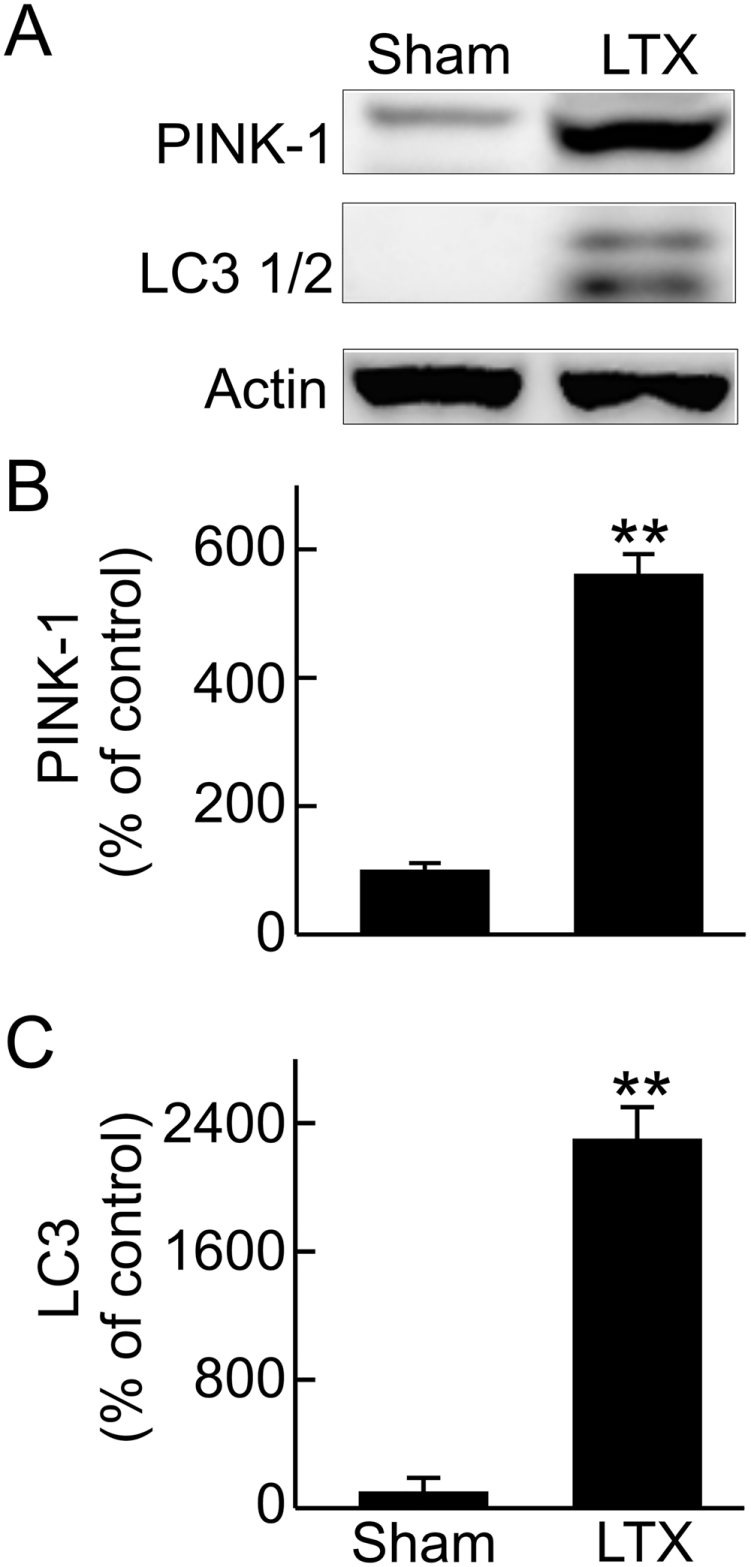
Increased renal mitophagy after liver transplantation. Transplantation was performed as described in “Methods” and kidneys were collected 18 h after sham-operation (**Sham**) or liver transplantation (**LTX**). **A**, representative immunoblot images of PTEN-induced putative kinase 1 (PINK-1) and microtubule-associated protein 1A/1B-light chain 3 (LC3); **B**, quantification of immunoblot images of PINK-1 by densitometry; **C**, quantification of immunoblot images of LC3 by densitometry. Values are mean ± SEM. **p<0.01 vs sham (n = 3-4/group).

Mitophagy removes damaged or aged mitochondria [29,30]. PINK-1 which regulates mitophagy [47,48] was expressed at low levels in the kidneys of sham-operated rats but increased 5.6-fold after LT (Fig 8A and 8B). LC3, a mediator of autophagy as well as mitophagy, increased 23-fold in the kidney after LT (Fig 8A and 8C). These data reveal that renal mitophagy is stimulated after LT.

## Discussion

AKI occurs frequently after LT, which decreases post-transplantation survival [7,10–13]. Therefore, understanding the mechanism and developing effective therapy for prevention and treatment of AKI is important for improvement of the clinical outcomes of LT. Renal dysfunction can occur in the presence (e.g. acute tubular necrosis, ATN) or absence of overt pathological changes (e.g. hepatorenal syndrome) [49–51]. The diagnosis of AKI currently depends on surrogate markers of kidney functions, such as increased serum creatinine and decreased urinary output [50–52]. Liver graft dysfunction is a strong predictor of AKI after LT, and combined liver failure and kidney failure substantially increases mortality [11,19,20]. In this study we show that transplantation of liver grafts after long cold-storage results in renal dysfunction and injury as revealed by increases in serum creatinine, BUN, renal NGAL expression and caspase-3 activation, and pathological changes.

The underlying mechanisms of AKI are poorly understood. In recent years emerging evidences indicate that mitochondrial dysfunction is an important contributor of AKI pathophysiology [21]. Mitochondrial ultrastructural changes such as decreased mitochondrial mass, disrupted cristae, and mitochondrial swelling occur in renal tubular cells during nephrotoxic, ischemic, and septic AKI [53–57]. Mitochondrial ultrastructural changes were observed in kidneys of hepatorenal syndrome patients [58]. Proper mitochondrial function requires maintaining mitochondrial homeostasis through removing damaged/depolarized mitochondria by mitophagy, synthesizing new mitochondrial components (e.g. OXPHOS proteins) and generating new mitochondria by MB, and maintaining mitochondrial dynamics [21,59,60]. Disruption of mitochondrial homeostasis is linked to AKI caused by many renal stressors/toxicants [21]. It is well known that chronic or acute severe liver injury/liver failure (e.g., cirrhosis, cholestasis, alcoholic or viral hepatitis, I/R injury and LT) causes AKI but whether mitochondrial homeostasis is disrupted in these cases remains unclear [61–66].

MB is an important adaptive process counteracting mitochondrial stress and damage (e.g. caused by toxicants and diseases) [23,67,68]. The majority of OXPHOS proteins are encoded by nDNA and transported into mitochondria after synthesis but 13 OXPHOS proteins are encoded by mtDNA and synthesized in mitochondria [68,69]. Recent studies showed that MB suppression occurs in many types of AKI [21]. In this study we demonstrate widespread decreases in renal OXPHOS proteins encoded by both nDNA and mtDNA in association with decreased corresponding mRNAs, indicating suppressed expression of OXPHOS proteins after LT. While sufficient mtDNA copy number is essential for MB and proper mitochondrial function [68], renal mtDNA decreased markedly (Fig 6), also indicating suppressed MB after LT. Together, these data demonstrate clearly that MB suppression also occurs in the kidney and contributes to post-LT AKI.

MB is coordinated by a complicated signaling system, and the transcriptional coactivator PGC-1α is recognized as the governing regulator of MB [70–73]. PGC-1α increases expression of nDNA-encoded OXPHOS enzymes and increases the expression of Tfam, which in turn controls the replication and transcription of mtDNA [70–73]. Therefore, PGC-1α also affects expression of mtDNA encoded OXPHOS proteins. Both expression of PGC-1α and its activation process (deacetylation) were inhibited in the kidney after LT. Consistently, Tfam and mtDNA were also diminished. Thus, suppressed MB after LT is due to inhibited PGC-1α signaling.

Mitophagy and mitochondrial dynamics are important processes that control mitochondrial quality [29,30,74]. Mitophagy occurs in response to starvation, loss of mitochondrial membrane potential or disruption of mitochondrial integrity [29,30]. Removal of damaged mitochondria via mitophagy in a timely manner is critical for cellular homeostasis and function [48,60]. Mitophagy occurs in a parkin-dependent and independent manner [48]. Mitochondria depolarization caused by various insults prevents degradation of PINK1, which in turn promotes parkin translocation to mitochondria. Parkin then enhances recruitment of autophagy receptor proteins (e.g., p62), which further recruit LC3 containing autophagosomes to execute mitophagy. For parkin-independent mitophagy, BNIP3, NIX, or cardiolipin directly interact with LC3 to recruit autophagosomes to remove damaged mitochondria [48,59]. Insufficient mitophagy has been associated with mitochondrial dysfunction and pathogenesis of many diseases such as Parkinson’s disease, cancer, cardiac dysfunction and hypertrophy, steatohepatitis, liver injury caused by hepatotoxicants, and inflammation [60]. However, overactive mitophagy may lead to mitochondrial loss and bioenergetic deficit, as in the case of hepatotoxicity of cadmium [75]. In the kidney, increased mitophagy has been observed in I/R- and sepsis-induced AKI and is thought to be renoprotective, possibly by removal of damaged mitochondria and mitochondria-generated ROS, and prevention of release of mtDNA and cytochrome c that cause inflammation and apoptosis, respectively [21,22,75,76]. In contrast, inhibited mitophagy was observed in high calorie diet-induced or hyperglycemic renal injury [31,77]. Our study showed that mitophagy is stimulated in the kidney after LT, which most likely reflects an attempt after LT to eliminate damaged mitochondria in the kidney.

Mitochondria undergo fission and fusion under physiological conditions [21,59]. Fission is thought to segregate damaged mitochondria which will be removed later, a process critical for mitochondrial quality control. Fission also participates in the process of MB to generate daughter mitochondria. Fusion allows the exchange of material between healthy and damaged mitochondria. Drp1 translocates from the cytosol to the outer mitochondrial membrane and interacts with receptor proteins in mitochondria (e.g., Fis1) to initiate fission [21,59]. Mitochondrial fusion is mediated by mitofusin 1 and 2 (Mfn1; Mfn2) and optic atrophy protein 1 [33,78]. Alterations of mitochondrial fission/fusion occur after I/R- and glycerol-induced AKI [22]. In this study we showed that after LT, Drp-1, Fis1 and Mfn1 all decreased in the kidney, indicating that fission and fusion processes in the kidney are suppressed after LT.

Taken together, MB and mitochondrial dynamics are inhibited in the kidney after LT and mitophagy increased. Such disruption of mitochondrial homeostasis decreases the capability of the kidney to counteract mitochondrial stress, maintain/recover mitochondrial function, and repair mitochondrial and cellular damage. Since renal tubular cells are highly mitochondrial energy-dependent, mitochondrial dysfunction will eventually lead to inhibition of renal function and renal cell damage. Based on these findings, stimulating MB to maintain mitochondrial homeostasis is a promising therapeutic target to prevent/treat AKI after LT. Stimulation of MB has been shown to be effective in several animal models of AKI and would most likely also be protective in AKI after LT.

## References

[1] Alqahtani SA. Update in liver transplantation. Curr Opin Gastroenterol. 2012; 10.1097/MOG.0b013e3283527f16 22450898

[2] Wertheim JA, Baptista PM, Soto-Gutierrez A. Cellular therapy and bioartificial approaches to liver replacement. Curr Opin Organ Transplant. 2012; 10.1097/MOT.0b013e3283534ec9 PMC3682785 22476224

[3] Harland RC, Platt JL. Prospects for xenotransplantation of the liver. J Hepatol. 1996; 25: 248–258. 8878790 10.1016/s0168-8278(96)80082-1

[4] Fox IJ, Langnas AN, Fristoe LW, Shaefer MS, Vogel JE, Antonson DL, et al Successful application of extracorporeal liver perfusion: a technology whose time has come. Am J Gastroenterol. 1993; 88: 1876–1881. 8237935

[5] Kobayashi N, Fujiwara T, Westerman KA, Inoue Y, Sakaguchi M, Noguchi H, et al Prevention of acute liver failure in rats with reversibly immortalized human hepatocytes. Science 2000; 287: 1258–1262. 10678831 10.1126/science.287.5456.1258

[6] Sampaio MS, Martin P, Bunnapradist S. Renal dysfunction in end-stage liver disease and post-liver transplant. Clin Liver Dis. 2014; 18: 543–560. S1089-3261(14)00031-2 [pii]; 10.1016/j.cld.2014.05.003 25017075

[7] Pham PT, Slavov C, Pham PC. Acute kidney injury after liver, heart, and lung transplants: dialysis modality, predictors of renal function recovery, and impact on survival. Adv Chronic Kidney Dis. 2009; 16: 256–267. S1548-5595(09)00080-9 [pii]; 10.1053/j.ackd.2009.04.002 19576556

[8] Biancofiore G, Davis CL. Renal dysfunction in the perioperative liver transplant period. Curr Opin Organ Transplant. 2008; 13: 291–297. 10.1097/MOT.0b013e328300a058 00075200-200806000-00014 [pii]. 18685320

[9] Nadim MK, Genyk YS, Tokin C, Fieber J, Ananthapanyasut W, Ye W, et al Impact of the etiology of acute kidney injury on outcomes following liver transplantation: acute tubular necrosis versus hepatorenal syndrome. Liver Transpl. 2012; 18: 539–548. 10.1002/lt.23384 22250075

[10] McCauley J, Van Thiel DH, Starzl TE, Puschett JB. Acute and chronic renal failure in liver transplantation. Nephron 1990; 55: 121–128. 2362625 10.1159/000185938 PMC2957102

[11] Weber ML, Ibrahim HN, Lake JR. Renal dysfunction in liver transplant recipients: evaluation of the critical issues. Liver Transpl. 2012; 18: 1290–1301. 10.1002/lt.23522 22847917

[12] O'Riordan A, Dutt N, Cairns H, Rela M, O'Grady JG, Heaton N, et al Renal biopsy in liver transplant recipients. Nephrol Dial Transplant. 2009; 24: 2276–2282. gfp112 [pii]; 10.1093/ndt/gfp112 19293134

[13] Barri YM, Sanchez EQ, Jennings LW, Melton LB, Hays S, Levy MF, et al Acute kidney injury following liver transplantation: definition and outcome. Liver Transpl. 2009; 15: 475–483. 10.1002/lt.21682 19399734

[14] Fraley DS, Burr R, Bernardini J, Angus D, Kramer DJ, Johnson JP. Impact of acute renal failure on mortality in end-stage liver disease with or without transplantation. Kidney Int. 1998; 54: 518–524. 10.1046/j.1523-1755.1998.00004.x 9690218

[15] Gonwa TA, McBride MA, Anderson K, Mai ML, Wadei H, Ahsan N Continued influence of preoperative renal function on outcome of orthotopic liver transplant (OLTX) in the US: where will MELD lead us? Am J Transplant. 2006; 6: 2651–2659. AJT1526 [pii]; 10.1111/j.1600-6143.2006.01526.x 16939515

[16] Zhu M, Li Y, Xia Q, Wang S, Qiu Y, Che M, et al Strong impact of acute kidney injury on survival after liver transplantation. Transplant Proc. 2010; 42: 3634–3638. S0041-1345(10)01302-3 [pii]; 10.1016/j.transproceed.2010.08.059 21094830

[17] Mehta RL. Outcomes research in acute renal failure. Semin Nephrol. 2003; 23: 283–294. S0270929503000640 [pii]. 12838497 10.1016/s0270-9295(03)00064-0

[18] Mehta RL, Chertow GM. Acute renal failure definitions and classification: time for change? J Am Soc Nephrol. 2003; 14: 2178–2187. 12874474 10.1097/01.asn.0000079042.13465.1a

[19] Cabezuelo JB, Ramirez P, Rios A, Acosta F, Torres D, Sansano T, et al Risk factors of acute renal failure after liver transplantation. Kidney Int. 2006; 69: 1073–1080. 5000216 [pii]; 10.1038/sj.ki.5000216 16528257

[20] Iglesias JI, DePalma JA, Levine JS. Risk factors for acute kidney injury following orthotopic liver transplantation: the impact of changes in renal function while patients await transplantation. BMC Nephrol. 2010; 11: 30 1471-2369-11-30 [pii]; 10.1186/1471-2369-11-30 21059264 PMC2991287

[21] Stallons LJ, Funk JA, Schnellmann RG. Mitochondrial Homeostasis in Acute Organ Failure. Curr Pathobiol Rep. 2013; 1 10.1007/s40139-013-0023-x PMC3874592 24386614

[22] Funk JA, Schnellmann RG. Persistent disruption of mitochondrial homeostasis after acute kidney injury. Am J Physiol Renal Physiol. 2012; 302: F853–F864. ajprenal.00035.2011 [pii]; 10.1152/ajprenal.00035.2011 22160772 PMC3340936

[23] Attardi G, Schatz G. Biogenesis of mitochondria. Annu Rev Cell Biol. 1988; 4: 289–333. 2461720 10.1146/annurev.cb.04.110188.001445

[24] Smith JA, Stallons LJ, Collier JB, Chavin KD, Schnellmann RG. Suppression of mitochondrial biogenesis through toll-like receptor 4-dependent mitogen-activated protein kinase kinase/extracellular signal-regulated kinase signaling in endotoxin-induced acute kidney injury. J Pharmacol Exp Ther. 2015; 352: 346–357. jpet.114.221085 [pii]; 10.1124/jpet.114.221085 25503387 PMC4293437

[25] Stallons LJ, Whitaker RM, Schnellmann RG. Suppressed mitochondrial biogenesis in folic acid-induced acute kidney injury and early fibrosis. Toxicol Lett. 2014; 224: 326–332. S0378-4274(13)01420-3 [pii]; 10.1016/j.toxlet.2013.11.014 24275386 PMC3987699

[26] Jesinkey SR, Funk JA, Stallons LJ, Wills LP, Megyesi JK, Beeson CC, et al Formoterol restores mitochondrial and renal function after ischemia-reperfusion injury. J Am Soc Nephrol. 2014; 25: 1157–1162. ASN.2013090952 [pii]; 10.1681/ASN.2013090952 24511124 PMC4033382

[27] Garrett SM, Whitaker RM, Beeson CC, Schnellmann RG. Agonism of the 5-hydroxytryptamine 1F receptor promotes mitochondrial biogenesis and recovery from acute kidney injury. J Pharmacol Exp Ther. 2014; 350: 257–264. jpet.114.214700 [pii]; 10.1124/jpet.114.214700 24849926 PMC4109485

[28] Korrapati MC, Shaner BE, Schnellmann RG. Recovery from glycerol-induced acute kidney injury is accelerated by suramin. J Pharmacol Exp Ther. 2012; 341: 126–136. jpet.111.190249 [pii]; 10.1124/jpet.111.190249 22228809 PMC3310704

[29] Kim I, Rodriguez-Enriquez S, Lemasters JJ. Selective degradation of mitochondria by mitophagy. Arch Biochem Biophys. 2007; 462: 245–253. S0003-9861(07)00162-2 [pii]; 10.1016/j.abb.2007.03.034 17475204 PMC2756107

[30] Lemasters JJ. Variants of mitochondrial autophagy: Types 1 and 2 mitophagy and micromitophagy (Type 3). Redox Biol. 2014; 2: 749–754. 10.1016/j.redox.2014.06.004 S2213-2317(14)00076-7 [pii]. 25009776 PMC4085350

[31] Gunst J, Derese I, Aertgeerts A, Ververs EJ, Wauters A, Van Den Berghe G, et al Insufficient autophagy contributes to mitochondrial dysfunction, organ failure, and adverse outcome in an animal model of critical illness. Crit Care Med. 2013; 41: 182–194. 10.1097/CCM.0b013e3182676657 23222264

[32] Liesa M, Palacin M, Zorzano A. Mitochondrial dynamics in mammalian health and disease. Physiol Rev. 2009; 89: 799–845. 89/3/799 [pii]; 10.1152/physrev.00030.2008 19584314

[33] Chan DC. Mitochondria: dynamic organelles in disease, aging, and development. Cell. 2006; 125: 1241–1252. S0092-8674(06)00768-9 [pii]; 10.1016/j.cell.2006.06.010 16814712

[34] Suen DF, Norris KL, Youle RJ. Mitochondrial dynamics and apoptosis. Genes Dev. 2008; 22: 1577–1590. 22/12/1577 [pii]; 10.1101/gad.1658508 18559474 PMC2732420

[35] Olichon A, Baricault L, Gas N, Guillou E, Valette A, Belenguer P, et al Loss of OPA1 perturbates the mitochondrial inner membrane structure and integrity, leading to cytochrome c release and apoptosis. J Biol Chem. 2003; 278: 7743–7746. 10.1074/jbc.C200677200 C200677200 [pii]. 12509422

[36] Liu Q, Rehman H, Shi Y, Krishnasamy Y, Lemasters JJ, Smith CD, et al Inhibition of sphingosine kinase-2 suppresses inflammation and attenuates graft injury after liver transplantation in rats. PLoS One 2012; 7: e41834 10.1371/journal.pone.0041834 PONE-D-12-14479 [pii]. 22848628 PMC3405047

[37] Zhong Z, Ramshesh VK, Rehman H, Currin RT, Sridharan V, Theruvath TP, et al Activation of the oxygen-sensing signal cascade prevents mitochondrial injury after mouse liver ischemia-reperfusion. Am J Physiol Gastrointest Liver Physiol. 2008; 295: G823–G832. 10.1152/ajpgi.90287.2008 18772364 PMC2575910

[38] Rehman H, Krishnasamy Y, Haque K, Thurman RG, Lemasters JJ, Schnellmann RG, et al Green tea polyphenols stimulate mitochondrial biogenesis and improve renal function after chronic cyclosporin a treatment in rats. PLoS One 2013; 8: e65029 10.1371/journal.pone.0065029 PONE-D-13-05193 [pii]. 23755172 PMC3670924

[39] Funk JA, Odejinmi S, Schnellmann RG. SRT1720 induces mitochondrial biogenesis and rescues mitochondrial function after oxidant injury in renal proximal tubule cells. J Pharmacol Exp Ther. 2010; 333: 593–601. jpet.109.161992 [pii]; 10.1124/jpet.109.161992 20103585 PMC2872958

[40] Puigserver P, Spiegelman BM. Peroxisome proliferator-activated receptor-gamma coactivator 1 alpha (PGC-1 alpha): transcriptional coactivator and metabolic regulator. Endocr Rev. 2003; 24: 78–90. 12588810 10.1210/er.2002-0012

[41] Pirola L, Zerzaihi O, Vidal H, Solari F. Protein acetylation mechanisms in the regulation of insulin and insulin-like growth factor 1 signalling. Mol Cell Endocrinol. 2012; 362: 1–10. S0303-7207(12)00305-X [pii]; 10.1016/j.mce.2012.05.011 22683437

[42] Goldstein SL. Acute kidney injury biomarkers: renal angina and the need for a renal troponin I. BMC Med. 2011; 9: 135 1741-7015-9-135 [pii]; 10.1186/1741-7015-9-135 22189039 PMC3287120

[43] Virbasius JV, Scarpulla RC. Activation of the human mitochondrial transcription factor A gene by nuclear respiratory factors: a potential regulatory link between nuclear and mitochondrial gene expression in organelle biogenesis. Proc Natl Acad Sci U S A. 1994; 91: 1309–1313. 8108407 10.1073/pnas.91.4.1309 PMC43147

[44] Dikov D, Reichert AS. How to split up: lessons from mitochondria. EMBO J. 2011; 30: 2751–2753. emboj2011219 [pii]; 10.1038/emboj.2011.219 21772324 PMC3160261

[45] Huang P, Galloway CA, Yoon Y. Control of mitochondrial morphology through differential interactions of mitochondrial fusion and fission proteins. PLoS One 2011; 6: e20655 10.1371/journal.pone.0020655 PONE-D-10-02872 [pii]. 21647385 PMC3103587

[46] Chen H, Detmer SA, Ewald AJ, Griffin EE, Fraser SE, Chan DC. Mitofusins Mfn1 and Mfn2 coordinately regulate mitochondrial fusion and are essential for embryonic development. J Cell Biol. 2003; 160: 189–200. 10.1083/jcb.200211046 jcb.200211046 [pii]. 12527753 PMC2172648

[47] Lazarou M, Jin SM, Kane LA, Youle RJ. Role of PINK1 binding to the TOM complex and alternate intracellular membranes in recruitment and activation of the E3 ligase Parkin. Dev Cell. 2012; 22: 320–333. S1534-5807(11)00580-6 [pii]; 10.1016/j.devcel.2011.12.014 22280891 PMC3288275

[48] Jin SM, Youle RJ. PINK1- and Parkin-mediated mitophagy at a glance. J Cell Sci. 2012; 125: 795–799. 125/4/795 [pii]; 10.1242/jcs.093849 22448035 PMC3656616

[49] Wadei HM, Gonwa TA. Hepatorenal Syndrome in the Intensive Care Unit. J Intensive Care Med. 2011; 0885066611408692 [pii]; 10.1177/0885066611408692 21859679

[50] Bagshaw SM, Bellomo R, Devarajan P, Johnson C, Karvellas CJ, Kutsiogiannis DJ, et al Acute kidney injury in critical illness. Can J Anaesth. 2010; 57: 985–998. 10.1007/s12630-010-9375-4 20931312

[51] Francoz C, Durand F. A new look at renal dysfunction in the cirrhotic patient. Crit Care 2012; 16: 118 cc11207 [pii]; 10.1186/cc11207 22385933 PMC3681343

[52] Bagshaw SM, Bellomo R, Devarajan P, Johnson C, Karvellas CJ, Kutsiogiannis DJ, et al Renal support in critical illness. Can J Anaesth. 2010; 57: 999–1013. 10.1007/s12630-010-9376-3 20931311

[53] Tran M, Tam D, Bardia A, Bhasin M, Rowe GC, Kher A, et al PGC-1alpha promotes recovery after acute kidney injury during systemic inflammation in mice. J Clin Invest. 2011; 121: 4003–4014. 58662 [pii]; 10.1172/JCI58662 21881206 PMC3195479

[54] Parikh SM, Yang Y, He L, Tang C, Zhan M, Dong Z. Mitochondrial function and disturbances in the septic kidney. Semin Nephrol. 2015; 35: 108–119. S0270-9295(15)00012-1 [pii]; 10.1016/j.semnephrol.2015.01.011 25795504 PMC4465453

[55] Brooks C, Wei Q, Cho SG, Dong Z. Regulation of mitochondrial dynamics in acute kidney injury in cell culture and rodent models. J Clin Invest. 2009; 119: 1275–1285. 37829 [pii]; 10.1172/JCI37829 19349686 PMC2673870

[56] Zsengeller ZK, Ellezian L, Brown D, Horvath B, Mukhopadhyay P, Kalyanaraman B, et al Cisplatin nephrotoxicity involves mitochondrial injury with impaired tubular mitochondrial enzyme activity. J Histochem Cytochem. 2012; 60: 521–529. 0022155412446227 [pii]; 10.1369/0022155412446227 22511597 PMC3460350

[57] Manny J, Livni N, Schiller M, Guttman A, Boss J, Rabinovici N. Structural changes in the perfused canine kidney exposed to the direct action of endotoxin. Isr J Med Sci. 1980; 16: 153–161. 7390755

[58] Mandal AK, Lansing M, Fahmy A. Acute tubular necrosis in hepatorenal syndrome: an electron microscopy study. Am J Kidney Dis. 1982; 2: 363–374. S0272638682000432 [pii]. 7148828 10.1016/s0272-6386(82)80096-6

[59] Ni HM, Williams JA, Ding WX. Mitochondrial dynamics and mitochondrial quality control. Redox Biol. 2015; 4: 6–13. S2213-2317(14)00118-9 [pii]; 10.1016/j.redox.2014.11.006 25479550 PMC4309858

[60] Redmann M, Dodson M, Boyer-Guittaut M, Darley-Usmar V, Zhang J. Mitophagy mechanisms and role in human diseases. Int J Biochem Cell Biol. 2014; S1357-2725(14)00168-X [pii]; 10.1016/j.biocel.2014.05.010 PMC4111979 24842106

[61] Choi HK, Song YG, Han SH, Ku NS, Jeong SJ, Baek JH, et al Clinical features and outcomes of acute kidney injury among patients with acute hepatitis A. J Clin Virol. 2011; 52: 192–197. S1386-6532(11)00288-5 [pii]; 10.1016/j.jcv.2011.07.013 21824812

[62] Bahirwani R, Shaked O, Bewtra M, Forde K, Reddy KR. Acute-on-chronic liver failure before liver transplantation: impact on posttransplant outcomes. Transplantation 2011; 92: 952–957. 10.1097/TP.0b013e31822e6eda 21869735

[63] Warner NS, Cuthbert JA, Bhore R, Rockey DC. Acute kidney injury and chronic kidney disease in hospitalized patients with cirrhosis. J Investig Med. 2011; 59: 1244–1251. 10.231/JIM.0b013e3182321471 21941210

[64] Lee HT, Park SW, Kim M, D'Agati VD. Acute kidney injury after hepatic ischemia and reperfusion injury in mice. Lab Invest. 2009; 89: 196–208. labinvest2008124 [pii]; 10.1038/labinvest.2008.124 19079326 PMC2632727

[65] Holt S, Marley R, Fernando B, Harry D, Anand R, Goodier D, et al Acute cholestasis-induced renal failure: effects of antioxidants and ligands for the thromboxane A2 receptor. Kidney Int. 1999; 55: 271–277. 10.1046/j.1523-1755.1999.00252.x 9893136

[66] Altamirano J, Fagundes C, Dominguez M, Garcia E, Michelena J, Cardenas A, et al Acute kidney injury is an early predictor of mortality for patients with alcoholic hepatitis. Clin Gastroenterol Hepatol. 2012; 10: 65–71. S1542-3565(11)01000-7 [pii]; 10.1016/j.cgh.2011.09.011 21946124

[67] Herrmann JM, Longen S, Weckbecker D, Depuydt M. Biogenesis of mitochondrial proteins. Adv Exp Med Biol. 2012; 748: 41–64. 10.1007/978-1-4614-3573-0_3 22729854

[68] Robin ED, Wong R. Mitochondrial DNA molecules and virtual number of mitochondria per cell in mammalian cells. J Cell Physiol. 1988; 136: 507–513. 3170646 10.1002/jcp.1041360316

[69] Koehler CM. New developments in mitochondrial assembly. Annu Rev Cell Dev Biol. 2004; 20: 309–335. 15473843 10.1146/annurev.cellbio.20.010403.105057

[70] Huss JM, Torra IP, Staels B, Giguere V, Kelly DP. Estrogen-related receptor alpha directs peroxisome proliferator-activated receptor alpha signaling in the transcriptional control of energy metabolism in cardiac and skeletal muscle. Mol Cell Biol. 2004; 24: 9079–9091. 15456881 10.1128/MCB.24.20.9079-9091.2004 PMC517878

[71] Winder WW, Taylor EB, Thomson DM. Role of AMP-activated protein kinase in the molecular adaptation to endurance exercise. Med Sci Sports Exerc. 2006; 38: 1945–1949. 17095928 10.1249/01.mss.0000233798.62153.50

[72] Lee HC, Wei YH. Mitochondrial biogenesis and mitochondrial DNA maintenance of mammalian cells under oxidative stress. Int J Biochem Cell Biol. 2005; 37: 822–834. 15694841 10.1016/j.biocel.2004.09.010

[73] Scarpulla RC. Nuclear activators and coactivators in mammalian mitochondrial biogenesis. Biochim Biophys Acta. 2002; 1576: 1–14. 12031478 10.1016/s0167-4781(02)00343-3

[74] Hoppins S. The regulation of mitochondrial dynamics. Curr Opin Cell Biol. 2014; 29C: 46–52. S0955-0674(14)00039-8 [pii]; 10.1016/j.ceb.2014.03.005 24747170

[75] Pi H, Xu S, Zhang L, Guo P, Li Y, Xie J, et al Dynamin 1-like-dependent mitochondrial fission initiates overactive mitophagy in the hepatotoxicity of cadmium. Autophagy 2013; 9: 1780–1800. 25665 [pii]; 10.4161/auto.25665 24121705

[76] Ishihara M, Urushido M, Hamada K, Matsumoto T, Shimamura Y, Ogata K, et al Sestrin-2 and BNIP3 regulate autophagy and mitophagy in renal tubular cells in acute kidney injury. Am J Physiol Renal Physiol. 2013; 305: F495–F509. ajprenal.00642.2012 [pii]; 10.1152/ajprenal.00642.2012 23698117

[77] Cui J, Shi S, Sun X, Cai G, Cui S, Hong Q, et al Mitochondrial autophagy involving renal injury and aging is modulated by caloric intake in aged rat kidneys. PLoS One 2013; 8: e69720 10.1371/journal.pone.0069720 PONE-D-13-01396 [pii]. 23894530 PMC3718786

[78] Hoppins S, Nunnari J. The molecular mechanism of mitochondrial fusion. Biochim Biophys Acta 2009; 1793: 20–26. S0167-4889(08)00255-3 [pii]; 10.1016/j.bbamcr.2008.07.005 18691613

